# Neural differences in processing of case particles in Japanese: an fMRI study

**DOI:** 10.1002/brb3.201

**Published:** 2013-12-31

**Authors:** Yosuke Hashimoto, Satoru Yokoyama, Ryuta Kawashima

**Affiliations:** 1 Department of Functional Brain Imaging, Institute of Development, Aging and Cancer, Tohoku UniversitySendai, Japan; 2 Faculty of International Liberal Arts, Akita International UniversityAkita, Japan

**Keywords:** Comprehension, functional MRI, language, neuroimaging, syntax

## Abstract

**Introduction:**

In subject–object–verb (SOV) languages, such as Japanese, sentence processing proceeds incrementally to the late presentation of the head (verb). Japanese case particles play a crucial role in sentence processing; however, little is known about how these particles are processed. In particular, it is still unclear how the functional difference between case particles is represented in the human brain.

**Methods:**

In this study, we conducted an fMRI experiment using an event-related design to directly compare brain activity during Japanese case particle processing among the nominative case *ga*, accusative case *o*, and dative case *ni*. Twenty five native Japanese speakers were asked to judge whether the presented character was a particle in a particle judgment task and whether the character ended with a specific vowel in a phonological judgment task, which was used as a control condition.

**Results:**

A particle comparison demonstrated that the processing of *ni* was associated with significantly weaker brain activity than that of *ga* and *o* in the left middle frontal gyrus (MFG) and the inferior frontal gyrus (IFG). Significantly greater brain activity associated with *ni* relative to *ga* in the right IFG was also observed.

**Conclusion:**

These results suggest that the Japanese case particles *ga*, *ni*, and *o* are represented differently in the brain.

## Introduction

More than 7000 languages currently exist in this world, and 43% of them are based on subject–object–verb (SOV) word order, including Japanese (Greenberg 1966; Lewis 2009). For example, the English sentence “Taro (S) read (V) a book (O)” is translated in Japanese as “Taro-ga (S) Hon-o (O) Yonda (V)” [Taro (S) a book (O) read (V)]. A sentence structure in SVO word order, like in English, can be determined at an earlier stage in the sentence because the head (verb) comes second in the order. By contrast, a sentence structure in the SOV word order, as in Japanese, cannot be recognized the same way because the head (verb) is not stated until the end of the sentence. These variations in language typology have been explored in psycholinguistic and cognitive processing models. Kamide (2006) and Yokoyama et al. (2012a) proposed a model in which Japanese sentences are incrementally processed before the head is inputted. Japanese has *ga* as the nominative marker, *o* as the accusative marker, and *ni* as the dative marker. Muraoka (2006) and Yasunaga et al. (2010) stated that the information contained in a case particle (e.g., *ni* or *o*) affects the prediction or anticipation of elements that will appear next. Therefore, the information contained in case particles plays a key role in the incremental process of interpreting the sentence before the verb appears. This difference between SOV languages and SVO languages is explored in a recent review of neuroimaging research (Hashimoto et al. 2012).

However, only a few studies have investigated the processing of case particles in the brain. Inui et al. (2007) examined the characteristics of case particle processing by showing participants case particles and non-case particles without any other sentence information (e.g., “X *ga*” (particle) or “X *nu*” (non-particle)) and asking them to judge whether it was a case particle or not. After comparing these results with those from a phonological task in which participants were required to judge whether the sound of *o* was included in a single Japanese character (hiragana) by using a block design, Inui et al. concluded that the left IFG is the region responsible for case particle processing in Japanese. Furthermore, Ogawa et al. (2007) and Ikuta et al. (2006) investigated the temporal dynamics of brain activity during sentence comprehension by analyzing stimulation when simple Japanese components are sequentially presented. Both studies reported left IFG activation during the stage of particle (or noun + particle) presentation. These results indicate that case particle processing is strongly associated with the left IFG. However, research on the neural representation of individual case particles is lacking.

In linguistic theory and language processing models, the nominative *ga*, accusative *o*, and dative *ni* are all categorized as grammatical cases, while only *ni* has functions that are different from *ga* and *o*. For example, *ni* works not only as a dative case marker but also as a semantic case marker (e.g., locative; Sadakane and Koizumi 1995). Hence, it has been assumed that *ni* shows different behavior from nominative *ga* and accusative *o* cases during sentence comprehension (Yokoyama et al. 2012a). Therefore, we predicted that the Japanese case particles *ga* and *o* would be associated with a similar pattern of brain activity, while *ni* would be associated with a different pattern. In order to test this hypothesis, we conducted a neuroimaging experiment designed to elucidate the processing differences among each Japanese case particle. The stimuli in Inui et al. (2007) were used in order to exclude the effect of nouns, verbs, or other sentential context.

## Material and Methods

### Participants

Thirty-three native speakers of Japanese (18 men and 15 women; aged 19–35 years; mean age = 22.3 years) participated in this study. All participants were right-handed, as confirmed by the Edinburgh Handedness Inventory (Oldfield 1971). None of the participants reported any previous history of medical diseases. Written informed consent was obtained from each subject in accordance with the guidelines of Tohoku University Medical School, and the Helsinki Declaration of Human Rights (1975). Eight participants’ data were excluded from analysis because of lower accuracy rates on target items (85% or lower on each target item used in the analysis [see Data Analysis]).

### Stimuli and task procedure

In this experiment, in order to set the context for a noun phrase, “X” followed by a single Japanese character (*hiragana*) was presented visually on a screen. Japanese is a head-final language in which a case is marked by a case particle system and all nouns are followed by case particles. In the hiragana writing system, the basic timing unit is called “mora,” and each mora takes equal time to pronounce. A single hiragana can represent a consonant and vowel or a vowel only. Target items were three case particles: *ga* (nominative case), *o* (accusative case), and *ni* (dative case). Non-particles were presented (“*u*,” “*nu*,” “*bu*,” “*za*,” “*ki*,” “*ro*”) as filler items.

The target experimental condition involved a particle judgment task in which participants were required to judge whether the character following “X” was a particle. This task was similar to that used in Inui et al. (2007). The control condition involved a phonological judgment task in which participants were required to judge whether the character following “X,” when spoken ended with the vowel sound [u]. In this task, participants were instructed to focus on only the phonological nature of the stimulus, so that activation associated with case particle processing could be determined by subtracting phonological judgment task-affiliated activation from particle judgment task-affiliated activation.

In this experiment, the particle and phonological judgment tasks were presented in different blocks. The tasks contained the same set of stimuli but differed with respect to the judgment of whether the stimulus indicated a Japanese particle or an [u]-ending letter. Each block contained 10 trials, which included five correct and five incorrect items. One session contained six blocks each, and participants were asked to perform two sessions. At the beginning of each block, participants saw the task instructions (“Particle” or “Phonological” in Japanese) for 1 sec.

The stimuli were presented visually on the screen inside the fMRI scanner for 1.5 sec followed by a fixation cross presentation for 3 sec. The inter-block interval was 10 sec. Participants were asked to judge which choice was correct by pressing buttons with their right hand. Trials were randomly presented within each block. The accuracy rates and response times for all tasks were collected using E-Prime software running on a Windows-based computer, which was also used for the visual presentation of experimental stimuli.

### Data acquisition

We collected fMRI scans using a 3T Intera Achieva MRI scanner (Achieva, Philips, Best, the Netherlands) at Tohoku University. Head motion was minimized by the use of cushions and tape around participants’ heads. Thirty axial slices (4 mm thickness; FOV = 192 mm; data matrix: 64 × 64 voxels) were acquired every 2 sec during functional measurements [BOLD-sensitive gradient EPI sequence; TR = 2000 msec; TE = 30 ms; flip angle = 70°]. Following functional image acquisition, anatomical T1-weighted images were also acquired from all participants.

### Data analysis

The fMRI time series data were analyzed using SPM5 software (Wellcome Trust Centre for Neuroimaging, http://www.fil.ion.ucl.ac.uk/) implemented on MATLAB (MathWorks, Inc., Shelborn, MA, USA). Slice-timing adjustment, realignment, spatial normalization to the standard brain space, and smoothing with an isotropic Gaussian kernel of 8-mm full width at half-maximum using the standard SPM method were carried out, and a high-pass frequency filter (128 sec) was applied. Time series data were modeled and convolved with the hemodynamic response function. Event-related analysis was performed. In the analysis, regressors of particle events, non-particle events, and incorrect responses were set in the first-level design. Particle events contained (1) *ga* in the particle judgment task (ga_par), (2) *ni* (ni_par), (3) *o* (o_par), (4) *ga* in the phonological judgment task (ga_pho), (5) *ni* (ni_pho) and (6) *o* (o_pho) as regressors of interest. Others were regressors of no interest. In the second-level analysis, the six images created in the first-level analysis were used to conduct the two-way ANOVA (task × particle) in order to determine whether previous results were replicated or not (e.g., main effect of task; Inui et al. 2007) and to examine whether different activations were observed among particles or not (task × particle interaction). For the brain activation data, group effects were computed using a random effects model, and the significance threshold was set at 0.001 (uncorrected for multiple comparisons). To control for false positives, we also adopted a cluster size limitation of >10 voxels (Forman et al. 1995). In addition to directly comparing conditions, we performed the post hoc ROI analysis based on mean beta values to explore how the detected regions represented differences among the task × particle interaction. We defined the significantly activated clusters in the comparisons as ROIs. Mean parameter estimates in each ROI for each subject within each condition were calculated. As we observed statistically significant differences in behavioral data among particles (see Results), we performed the ROI analysis using the ANCOVA with behavioral data as a covariate to test whether observed brain activity was affected by behavioral differences. In addition, post hoc multiple comparisons were performed (Bonferroni correction).

## Results

### Behavioral data

Table 1 summarizes accuracy rates and reaction times (RTs). Accuracy rates did not differ significantly between the particle judgment task and the phonological judgment task and among the three particles as analyzed by the two-way repeated-measures ANOVA (rANOVA) [task: *F*_1,24_ = 0.325, *P* = 0.574; particle: *F*_2,23_ = 1.944, *P* = 0.166]. Analysis of RTs using the two-way rANOVA revealed a main effect of particle, but no significant difference between the particle judgment task and the phonological judgment task [task: *F*_1,24_ = 1.602, *P* = 0.218; particle: *F*_2,23_ = 6.532, *P* = 0.003]. The post hoc test showed that the RTs for *ga* were significantly shorter than those for the other particles (Bonferroni, *P* < 0.05, “*ga* < *ni*,” “*ga* < *o*”).

**Table 1:** Behavioral data for target conditions

Task	Particle	Accuracy (%)	SD	RTs (ms)	SD
Particle	*ga*	98.6	3.4	534.0	71.9
	*ni*	97.8	2.7	648.3	288.5
	*o*	97.6	3.6	664.8	246.5
Phonology	*ga*	99.0	2.5	592.0	150.2
	*ni*	98.4	3.5	678.6	212.3
	*o*	97.7	3.7	725.2	277.6

Reaction times (RTs) were obtained from trials with corrected responses.

### Imaging data

**Figure 1:**
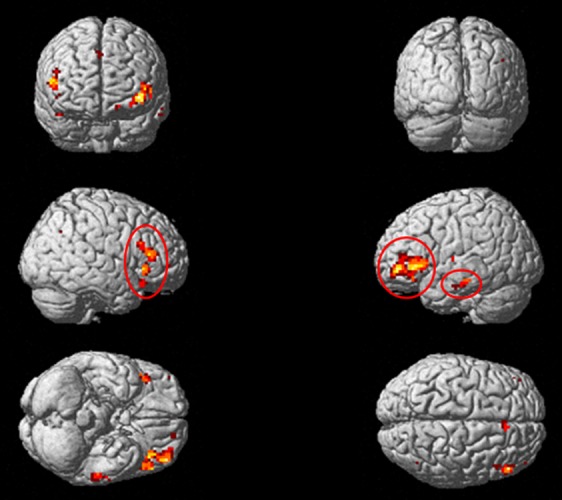
Brain activity associated with the Particle Judgment task. Results of a whole-brain analysis using the two-way ANOVA are shown. The significant activations are projected onto a rendered brain surface in MNI stereotactic space. The contrasts of these results were a positive main effect of task (particle > phonological). The significance threshold was set at *P* < 0.001, uncorrected. Areas that showed cluster size *k* > 10 are circled with a red line.

**Figure 2:**
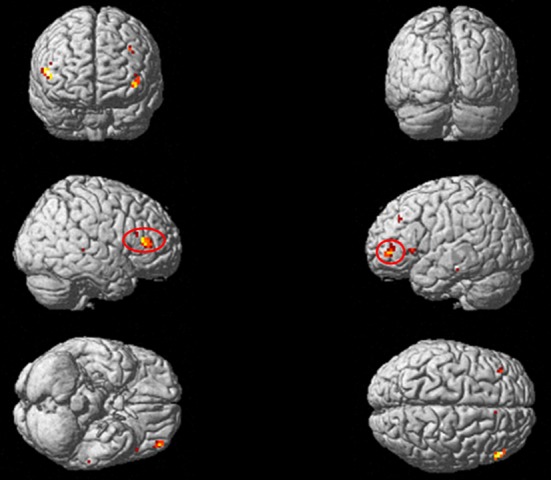
Brain activity associated with case particles. Results of a whole-brain analysis using the two-way ANOVA are shown. The significant activations are projected onto a rendered brain surface in MNI stereotactic space. The contrasts of these results were a significant interaction: ([*ga* in particle task > *ga* in phonological task] vs. [*ni* in particle task > *ni* in phonological task] vs. [*o* in particle task > *o* in phonological task]). The significance threshold was set at *P* < 0.001, uncorrected. Areas that showed cluster size *k* > 10 are circled with a red line.

Results showed greater activity in the middle frontal gyrus (MFG), the right inferior frontal gyrus (IFG), and the left inferior temporal gyrus (ITG) during the particle task than the phonological task (Fig. 1 and Table 2). Significantly greater activation was not associated with *ga*, *ni*, and *o* during the phonological judgment task than the particle judgment task. Next, we tested for specific areas of brain activity associated with case particle processing. We performed the ANOVA to assess a potential task × particle interaction ([*ga* in particle task > *ga* in phonological task] vs. [*ni* in particle task > *ni* in phonological task] vs. [o in particle task > o in phonological task]). Results showed that each of the three types of case particle processing were associated with different patterns of activity in the left MFG and the right and left IFG (Table 3 and Fig. 2).

**Table 2:** Imaging results for a positive effect of particle task

Anatomical label	Cluster level corrected	Cluster size	*T*	*Z*	*x*	*y*	*z*
Left hemisphere							
Inferior frontal gyrus	0.001	201	4.15	4.03	−48	21	0
Middle frontal gyrus			3.94	3.84	−42	48	−3
Inferior frontal gyrus			3.85	3.75	−51	30	3
Inferior temporal gyrus	0.569	22	4.25	4.12	−60	−27	−18
			3.27	3.21	−63	−12	−18
Right hemisphere							
Superior frontal gyrus	0.692	17	4.01	3.9	6	30	42
Inferior frontal gyrus	0.032	89	3.99	3.88	51	33	12
			3.59	3.51	48	24	21
			3.96	3.85	42	27	−3
Inferior frontal gyrus	0.818	12	3.56	3.58	45	24	−18

Results of a whole-brain analysis of a positive main effect of task (particle > phonological) from the two-way ANOVA are shown. The significance threshold was set at *P* < 0.001, uncorrected, and cluster size was *k* > 10.

**Table 3:** Imaging results of a task × particle interaction

ROI	Anatomical label	Cluster size	*F*	*Z*	*x*	*y*	*z*
Left hemisphere							
a	Middle frontal gyrus	20	6.82	3.48	−42	51	−3
	Inferior frontal gyrus pars triangularis		6.21	3.27	−45	45	6
Right hemisphere							
b	Inferior frontal gyrus pars triangularis	28	8.12	3.90	54	33	12

This table shows results of a task × particle interaction: ([*ga* in particle task > *ga* in phonological task] vs. [*ni* in particle task > *ni* in phonological task] vs. [*o* in particle task > *o* in phonological task]) from the two-way ANOVA. The statistical threshold was set at *P* < 0.001, uncorrected, and cluster size *k* > 10.

### ROI analysis

**Figure 3:**
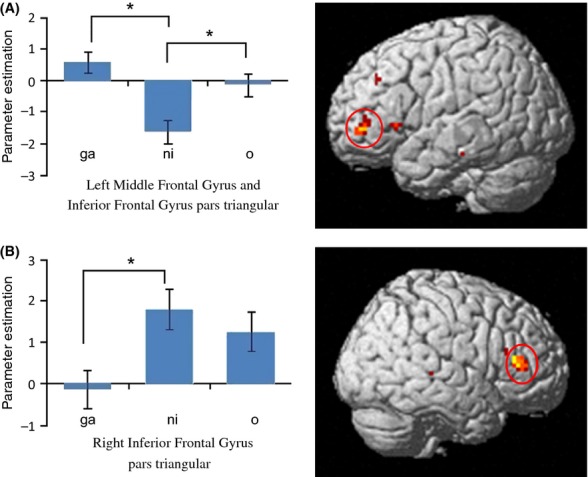
ROI analysis of case particles. These figures show results of the two-way ANCOVA with RTs as a covariate in order to exclude the effects of reaction times on each ROI. Panels “A” and “B” correspond, respectively, to Table 3. The blue bars in the graphs show the parameter estimates of beta values in each contrast. Asterisks indicate a statistically significant difference (*P* < 0.05, Bonferroni). Case particles “*ga*,” “*ni*,” and “*o*” in the figure represent the target contrasts, “*ga* in particle task > *ga* in phonological task,” “*ni* in particle task > *ni* in phonological task,” and “*o* in particle task > *o* in phonological task,” respectively.

We conducted the post hoc ROI analysis for the two regions of the brain (“a” and “b” in Table 3). Results of this analysis are shown in Figure 3. In the left MFG and left IFG ROIs, significantly greater brain activity was associated with “*ga*” and “*o*” relative to “*ni*” [Bonferroni, *ga* > *ni*: *P* = 0.000; *o* > *ni*: *P* = 0.021]. In the right IFG, brain activity associated with “*ni*” was significantly higher than that of “*ga*” [IFG: *P* = 0.016].

## Discussion

The aim of this study was to investigate whether and how the processing of individual case particles (nominative case *ga*, accusative case *o*, and dative case *ni*) is represented in the human brain. Significantly greater activity in the left MFG and left IFG was associated with *ga* and *o* relative to *ni* (Fig. 3). In addition, greater activity in the right IFG was associated with *ni* relative to *ga*. Our results indicate that the case particles *ga*, *o*, and *ni* are processed differently in the human brain.

In addition to our main conclusion, at least three alternative explanations are possible. First, it is necessary to confirm that our experimental stimuli appropriately assessed case particle processing. The strongest indication for this possibility is the significant positive effect of the particle judgment task associated with the left IFG that has been reported in previous studies (Ikuta et al. 2006; Inui et al. 2007; Ogawa et al. 2007) using the same experimental design and hypothesis (see Data Analysis, Table 2 and Fig. 1). Although additional regions were associated with the stimuli in our experiment (Table 2, Figure 1), the largest cluster was mainly located within the left IFG. Furthermore, the other regions are commonly known to play a role in language (e.g., Yokoyama et al. 2006, 2007, 2012b; Price 2010). Therefore, it is likely that our experiment assessed case particle processing.

Second, the observed imaging data in this study may be affected by the behavioral data obtained. The RTs differed significantly among particles (see Results and Table 1). However, this finding cannot explain all brain activation patterns. The RTs for *ga* were significantly smaller than those for *ni* and *o*. Given that this difference was observed in both the particle and phonological tasks, it is plausible that the difference was due to the *hiragana* effect rather than particle processing. Unfortunately, no previous studies have explored individual differences in the rate of Japanese hiragana reading. However, as we performed ROI analyses by using the ANCOVA with RTs as a covariate, the signal changes found in our analysis (Fig. 3) cannot be explained by RT differences. Therefore, the behavioral data observed in this study did not account for our brain activity results.

Finally, the *hiragana* effect should be considered. We suggested that the difference in RTs resulted from individual differences in the rate of hiragana letter reading. However, we compared the particle task and the phonological task to eliminate the hiragana effect (see Material and Methods). Consequently, any hiragana-mediated effects on RTs did not influence the signal change results in the case particle effect.

The aforementioned evidence leads us to conclude that the observed differences in brain activity did not result from factors other than the differences in case particles. Finally, we would like to discuss how the brain processes case particles. As predicted on the basis of previous studies, significantly weaker brain activity was associated with *ni* relative to *ga* and *o* in the left MFG (Brodmann area 46: BA46) (Table 3) and the IFG pars triangularis (Brodmann area 45: BA45; Fig. 3). BA45 has been implicated in syntactic processing (e.g., Just et al. 1996; Hashimoto and Sakai 2002; Friederici et al. 2003; Fiebach et al. 2005; Yokoyama et al. 2006, 2007). It is possible that this finding supports theory delineated in the Introduction (i.e., that *ga* and *o* are grammatical cases while *ni* has various functions, and is thus less specific to syntactic processing). We also observed significantly greater brain activity associated with *ni* relative to *ga* in the right IFG. Currently, it remains unclear why such patterns were observed, but one possibility is that these brain regions mediate dative and accusative case processing in Japanese.

## Conclusion

We conducted an fMRI experiment to investigate differences in brain activity during Japanese case particle processing among the nominative case *ga*, accusative case *o*, and dative case *ni*. The comparison among particles showed that brain activity associated with *ni* was significantly weaker than that of *ga* and *o* in the left MFG and left IFG. Furthermore, significantly greater brain activity was associated with *ni* relative to *ga* in the right IFG. These findings suggest that the Japanese case particles *ga*, *ni*, and *o* are represented differently in the brain. As we used stimuli that lacked nouns or verbs, this study is limited to case particle processing. Therefore, our findings indicate that individual case particles have a distinct neural representation, and consequently, might play disparate roles in language processing.
